# Notification that new names of prokaryotes, new combinations and new taxonomic opinions have appeared in volume 76, part 7 of the IJSEM

**DOI:** 10.1099/ijsem.0.007280

**Published:** 2026-10-29

**Authors:** Aharon Oren, Markus Göker

**Affiliations:** 1 The Institute of Life Sciences, The Hebrew University of Jerusalem, The Edmond J. Safra Campus, 9190401 Jerusalem, Israel; 2 Leibniz Institute DSMZ – German Collection of Microorganisms and Cell Cultures, Inhoffenstrasse 7B, 38124 Braunschweig, Germany

This listing of names of prokaryotes published in a previous issue of the *International Journal of Systematic and Evolutionary Microbiology* (*IJSEM*) is provided as a service to microbiology to assist in the recognition of new names and new combinations. This procedure was proposed by the Judicial Commission [1]. The names given herein are listed according to the Rules of priority (i.e. time and date of publication of the articles on the journal’s platform and the order of valid publication of names in the original articles) [2].

**Table:** 

Order of article publication	Name/authors	Proposed as	Article no.	Page no.
1	*Staphylococcus dromedarii* Kuhnert *et al*. 2026	sp. nov.	007230	8
2	*Leucogemmata* Suzuki *et al*. 2026	gen. nov.	007204	9
2	*Leucogemmata humicola* Suzuki *et al*. 2026	sp. nov.	007204	9
3	*Nocardia glycosmidis* Sripreechasak *et al*. 2026	sp. nov.	007218	8
3	*Nocardia aegles* Sripreechasak *et al*. 2026	sp. nov.	007218	9
3	*Nocardia citri* Sripreechasak *et al*. 2026	sp. nov.	007218	9
4	*Streptococcus intestinihominis* Li *et al*. 2026	sp. nov.	007210	8
5	*Caldifarcimen* Mori *et al*. 2026	gen. nov.	007229	8
5	*Caldifarcimen microaerophilum* Mori *et al*. 2026	sp. nov.	007229	9
5	*Caldifarciminaceae* Mori *et al*. 2026	fam. nov.	007229	9
5	*Caldifarciminales* Mori *et al*. 2026	ord. nov.	007229	9
5	*Caldifarciminia* Mori *et al*. 2026	class. nov.	007229	9
5	*Caldifarciminota* Mori *et al*. 2026	phyl. nov.	007229	9
6	*Sulfurimonas lacustris* Watanabe *et al*. 2026	sp. nov.	007228	6
6	*Sulfurimonas limnophila* Watanabe *et al*. 2026	sp. nov.	007228	7
7	*Neoluteolibacter* Dali *et al*. 2026	gen. nov.	007231	11
7	*Neoluteolibacter arcticus* (Kim *et al*. 2015) Dali *et al*. 2026	comb. nov. [basonym: *Luteolibacter arcticus* Kim *et al*. 2015]	007231	11
7	*Neoluteolibacter flavescens* (Zhang *et al*. 2017) Dali *et al*. 2026	comb. nov. [basonym: *Luteolibacter flavescens* Zhang *et al*. 2017]	007231	11
7	*Neoluteolibacter luteus* (Dahal *et al*. 2021) Dali *et al*. 2026	comb. nov. [basonym: *Luteolibacter luteus* Dahal *et al*. 2021]	007231	11
7	*Neoluteolibacter rhizosphaerae* (Shen *et al*. 2024) Dali *et al*. 2026	comb. nov. [basonym: *Luteolibacter rhizosphaerae* Shen *et al*. 2024]	007231	12
7	*Neoluteolibacter marinus* (Xie *et al*. 2022) Dali *et al*. 2026	comb. nov. [basonym: *Luteolibacter marinus* Xie *et al*. 2022]	007231	12
7	*Neoluteolibacter soli* Dali *et al*. 2026	sp. nov.	007231	12
7	‘*Candidatus* Parakkermansia’ Dali *et al*. 2026^*^	gen. nov.	007231	12
7	‘*Candidatus* Parakkermansia intestinavium’ (Gilroy *et al*. 2021) Dali *et al*. 2026*	comb. nov. [basonym: ‘*Candidatus* Akkermansia intestinavium’ Gilroy *et al*. 2021]	007231	12
7	‘*Candidatus* Parakkermansia intestinigallinarum’ (Gilroy *et al*. 2021) Dali *et al*. 2026*	comb. nov. [basonym: ‘*Candidatus* Akkermansia intestinigallinarum’ Gilroy *et al*. 2021]	007231	13
7	*Neoakkermansia* Dali *et al*. 2026	gen. nov.	007231	13
7	*Neoakkermansia glycaniphila* (Ouwerkerk *et al*. 2016) Dali *et al*. 2026	comb. nov. [basonym: *Akkermansia glycaniphila* Ouwerkerk *et al*. 2016]	007231	13
7	*Prosthecobacter gellanilyticus* (Otsuka *et al*. 2013) Dali *et al*. 2026	comb. nov. [basonym: *Brevifollis gellanilyticus* Otsuka *et al*. 2013]	007231	13
8	*Porcibacter* Paek *et al*. 2026	gen. nov.	007214	7
8	*Porcibacter vitabionis* Paek *et al*. 2026	sp. nov.	007214	7
9	*Pseudoalteromonas mucoides* Kim *et al*. 2026	sp. nov.	007235	10
9	*Pseudoalteromonas aquimarina* Kim *et al*. 2026	sp. nov.	007235	10
9	*Pseudoalteromonas maris* Kim *et al*. 2026	sp. nov.	007235	11
9	*Pseudoalteromonas thalassia* Kim *et al*. 2026	sp. nov.	007235	11
10	*Mycobacterium toneyamaense* Komine *et al*. 2026	sp. nov.	007219	6
11	*Pasteurella pantherarum* Christensen *et al*. 2026	sp. nov.	007232	7
12	*Sinomicrobium endophyticum* Sun *et al*. 2026	sp. nov.	007240	7
12	*Sinomicrobium halophytocola* Sun *et al*. 2026	sp. nov.	007240	7
13	*Pseudooceanicola profundus* Zhu *et al*. 2026	sp. nov.	007237	8
14	*Celerinatantimonas sediminis* Du *et al*. 2026	sp. nov.	007155	7
14	*Celerinatantimonas marina* Du *et al*. 2026	sp. nov.	007155	7
15	*Anaeromusa burkinensis* corrig. (Ouattara *et al*. 1992) Heng 2026	comb. nov. [basonym: *Anaerovibrio burkinensis* corrig. Ouattara *et al*. 1992]	007239	7
15	*Thermosinus thermophilus* (Ogg and Patel 2009) Heng 2026	comb. nov. [basonym: *Sporolituus thermophilus* Ogg and Patel 2009]	007239	7
15	‘*Pelosinus colonicu*s’ Heng 2026^§^	sp. nov. [earlier, not validly published, homotypic synonym: ‘*Sporotalea colonica*’ Ezaki *et al*. 2008]	007239	7
16	*Azospirillum caeni* Wan *et al*. 2026	sp. nov.	007227	8
17	*Marinibium* Lee *et al*. 2026	gen. nov.	007217	8
17	*Marinibium conchae* Lee *et al*. 2026	sp. nov.	007217	8
18	*Neffella* Silva Cerqueira *et al*. 2026	gen. nov.	007119	14
18	*Neffella xylocopae* Silva Cerqueira *et al*. 2026	sp. nov.	007119	14
19	*Pseudarthrobacter ribosi* Yang *et al*. 2026	sp. nov.	007242	7
19	*Pseudarthrobacter leeuwenhoeki* Yang *et al*. 2026	sp. nov.	007242	7
19	*Pseudarthrobacter linnaei* Yang *et al*. 2026	sp. nov.	007242	8
19	*Pseudarthrobacter adonitolis* Yang *et al*. 2026	sp. nov.	007242	8
19	*Pseudarthrobacter kochi* Yang *et al*. 2026	sp. nov.	007242	8
19	*Pseudarthrobacter woesei* Yang *et al*. 2026	sp. nov.	007242	9
19	*Pseudarthrobacter xylosi* Yang *et al*. 2026	sp. nov.	007242	9
19	*Pseudarthrobacter lactosi* Yang *et al*. 2026	sp. nov.	007242	10
19	*Pseudarthrobacter arbutini* Yang *et al*. 2026	sp. nov.	007242	10
20	*Paenibacillus tabacirhizosphaerae* Liu *et al*. 2026	sp. nov.	007215	10
21	*Marinomonas algae* Moon *et al*. 2026	sp. nov.	007247	9
21	*Marinomonas phycocola* Moon *et al*. 2026	sp. nov.	007247	9
22	*Erythrobacter epiphyticus* Maltman *et al*. 2026	sp. nov.	007252	8
22	*Sphingomonas epibiotica* Maltman *et al*. 2026	sp. nov.	007252	9
23	*Patulibacter endophyticus* Lu *et al*. 2026	sp. nov.	007248	8
24	*Arthrobacter glycogeni* Yang *et al*. 2026	sp. nov.	007246	7
24	*Arthrobacter arabinosi* Yang *et al*. 2026	sp. nov.	007246	7
24	*Arthrobacter melezitosi* Yang *et al*. 2026	sp. nov.	007246	8
24	*Arthrobacter sorbitolis* Yang *et al*. 2026	sp. nov.	007246	8
24	*Arthrobacter rhamnosi* Yang *et al*. 2026	sp. nov.	007246	8
24	*Arthrobacter pasteuri* Yang *et al*. 2026	sp. nov.	007246	9
25	*Jujubicoccus* Li *et al*. 2026	gen. nov.	007244	8
25	*Jujubicoccus corallicola* Li *et al*. 2026	sp. nov.	007244	8
25	*Agaricicolaceae* Li *et al*. 2026	fam. nov.	007244	9
26	*Treponema pallidum* subsp. *pallidum* (Schaudinn and Hoffmann 1905) Norris *et al*. 2026^†^	comb. nov. [basonym: ‘*Spirochaeta pallida*’ Schaudinn and Hoffmann 1905]	007245	12
26	*Treponema pallidum* subsp. *pertenue* (Castellani 1905) Norris *et al*. 2026^†^	comb. nov. [basonym: ‘*Spirochaeta pertenuis*’ Castellani 1905]	007245	12
26	*Treponema pallidum* subsp. *endemicum* Norris *et al*. 2026^‡^	subsp. nov.	007245	12
27	*Testudinibacter crocodili* Ronco *et al*. 2026	sp. nov.	007236	6
28	*Streptomyces borealis* Biessy *et al*. 2026	sp. nov.	007254	11
28	*Streptomyces saintjeani* Biessy *et al*. 2026	sp. nov.	007254	11
29	*Gorillibacterium terrae* Liu *et al*. 2026	sp. nov.	007259	9
29	*Oryzibacillus* Liu *et al*. 2026	gen. nov.	007259	9
29	*Oryzibacillus soli* Liu *et al*. 2026	sp. nov.	007259	9
30	*Pacificoceanicola niger* Wang *et al*. 2026	sp. nov.	007251	6
30	*Pacificoceanicola algae* Wang *et al*. 2026	sp. nov.	007251	6
30	*Pacificoceanicola oceani* (Zhou *et al*. 2025) Wang *et al*. 2026	comb. nov. [basonym: *Tropicibacter oceani* Zhou *et al*. 2025]	007251	6

*Name pro-validly published.

§Name neither pro-validly published nor validly published. If the designated type strain culture was suitable for publication, it should have been deposited in at least two culture collections in different countries. If the culture was demonstrably unsuitable for publication, the nomenclatural type could be a nucleotide sequence; however, no such demonstration was made.

†The organism was assigned to Risk Group 2 [German TRBA 466, ‘Einstufung von Prokaryonten (Bacteria und Archaea) in Risikogruppen’].

‡The species for which the new subspecies was described was assigned to Risk Group 2 [German TRBA 466, ‘Einstufung von Prokaryonten (Bacteria und Archaea) in Risikogruppen’].

The following taxonomic opinions (i.e. the emendation of circumscriptions or the creation of synonyms) do not affect the status of a name under the International Code of Nomenclature of Prokaryotes. These taxonomic opinions can neither be considered as approved nor as disapproved by the International Committee on Systematics of Prokaryotes.

**Table:** 

Name/authors:	Article no.	Page no.
*Akkermansia* Derrien *et al*. 2004 emend. Dali *et al*. 2026	007231	12
*Anaeroarcus* Strömpl *et al*. 1999 pro synon. *Anaeromusa* Baena *et al*. 1999	007239	3
*Anaeromusa acidaminophila* Baena *et al*. 1999 pro synon. *Anaeromusa burkinensis* corrig. (Ouattara *et al*. 1992) Heng 2026	007239	3
*Arthrobacter vasquezii* Valenzuela-Ibaceta *et al*. 2023 pro synon. *Arthrobacter parietis* Heyrman *et al*. 2005	007246	7
*Barrientosiimonas endolithica* Parag *et al*. 2015 pro synon. *Barrientosiimonas humi* Lee *et al*. 2013	007241	6
*Barrientosiimonas humi* Lee *et al*. 2013 emend. Komaki 2026	007241	6
*Dermacoccus abyssi* Pathom-aree *et al*. 2006 emend. Komaki 2026	007241	6
*Dermacoccus barathri* Pathom-aree *et al*. 2006 pro synon. *Dermacoccus abyssi* Pathom-aree *et al*. 2006	007241	6
*Dermacoccus profundi* Pathom-aree *et al*. 2006 pro synon. *Dermacoccus abyssi* Pathom-aree *et al*. 2006	007241	6
*Pacificoceanicola* Yang *et al*. 2023 emend. Wang *et al*. 2026	007251	6
*Paracoccaceae* Liang *et al*. 2022 emend. Li *et al*. 2026	007244	9
*Prosthecobacter* (ex Staley *et al*. 1976) Staley *et al*. 1980 emend. Dali *et al*. 2026	007231	13
*Roseobacteraceae* Liang *et al*. 2021 emend. Li *et al*. 2026	007244	9
*Rubritalea halochordaticola* Yoon *et al*. 2011 pro synon. *Rubritalea squalenifaciens* Kasai *et al*. 2007*	007231	14
*Sporolituus* Ogg and Patel 2009 pro synon. *Thermosinus* Sokolova *et al*. 2004	007239	5
*Sporomusa paucivorans* Hermann *et al*. 1987 pro synon. *Sporomusa sphaeroides* Möller *et al*. 1985	007239	2
*Treponema pallidum* (Schaudinn and Hoffmann 1905) Schaudinn 1905 (Approved Lists 1980) emend. Norris *et al*. 2026^†^	007245	11
*Verrucomicrobiaceae* Ward-Rainey *et al*. 1996 emend. Dali *et al*. 2026	007231	10
*Yimella lutea* Tang *et al*. 2010 emend. Komaki 2026	007241	6
*Yimella radicis* Yang *et al*. 2016 pro synon. *Yimella lutea* Tang *et al*. 2010	007241	6

*The authors gave *R. halochordaticola* Yoon *et al. *2011 pro synon. *R. squalenifaciens* Kasai *et al. *2007.

†The organism was assigned to Risk Group 2 [German TRBA 466, ‘Einstufung von Prokaryonten (Bacteria und Archaea) in Risikogruppen’].

## Corrigendum

The name *Mycobacteroides stephanolepidis* (Fukano *et al*. 2017) Val-Calvo *et al*. 2025 listed in the Notification List for volume 75, part 9 [3], is not validly published because the type strain deposits listed were not those of the basonym, although the name was proposed as a new combination. The published correction [4] is useless because it does not include a complete protologue.
